# The Positive Allosteric Modulation of alpha7-Nicotinic Cholinergic Receptors by GAT107 Increases Bacterial Lung Clearance in Hyperoxic Mice by Decreasing Oxidative Stress in Macrophages

**DOI:** 10.3390/antiox10010135

**Published:** 2021-01-19

**Authors:** Alex G. Gauthier, Jiaqi Wu, Mosi Lin, Ravikumar Sitapara, Abhijit Kulkarni, Ganesh A. Thakur, Edward E. Schmidt, Jeanette C. Perron, Charles R. Ashby, Lin L. Mantell

**Affiliations:** 1 Department of Pharmaceutical Sciences, College of Pharmacy and Health Sciences, St. John’s University, Queens, New York, NY 11439, USA; Alexgauthier4@gmail.com (A.G.G.); Jiaqi.wu16@stjohns.edu (J.W.); mosi.lin19@stjohns.edu (M.L.); Ravi.sitapara@gmail.com (R.S.); perronj@stjohns.edu (J.C.P.); cnsratdoc@optonline.net (C.R.A.J.); 2 Department of Pharmaceutical Sciences, Northeastern University, Boston, MA 02115, USA; kulkarni.abh@husky.neu.edu (A.K.); g.thakur@northeastern.edu (G.A.T.); 3 Department of Microbiology and Immunology, Montana State University, Bozeman, MT 59717, USA; eschmidt@montana.edu; 4 Feinstein Institute for Medical Research, Northwell Health, Manhasset, NY 11030, USA

**Keywords:** hyperoxia, α7nAChR, GAT107, ago-PAM, macrophage, pulmonary infection, antioxidant, oxygen therapy, phagocytosis, vagus nerve, oxidative stress

## Abstract

Supplemental oxygen therapy with supraphysiological concentrations of oxygen (hyperoxia; >21% O_2_) is a life-saving intervention for patients experiencing respiratory distress. However, prolonged exposure to hyperoxia can compromise bacterial clearance processes, due to oxidative stress-mediated impairment of macrophages, contributing to the increased susceptibility to pulmonary infections. This study reports that the activation of the α7 nicotinic acetylcholine receptor (α7nAChR) with the delete allosteric agonistic-positive allosteric modulator, GAT107, decreases the bacterial burden in mouse lungs by improving hyperoxia-induced lung redox imbalance. The incubation of RAW 264.7 cells with GAT107 (3.3 µM) rescues hyperoxia-compromised phagocytic functions in cultured macrophages, RAW 264.7 cells, and primary bone marrow-derived macrophages. Similarly, GAT107 (3.3 µM) also attenuated oxidative stress in hyperoxia-exposed macrophages, which prevents oxidation and hyper-polymerization of phagosome filamentous actin (F-actin) from oxidation. Furthermore, GAT107 (3.3 µM) increases the (1) activity of superoxide dismutase 1; (2) activation of Nrf2 and (3) the expression of heme oxygenase-1 (HO-1) in macrophages exposed to hyperoxia. Overall, these data suggest that the novel α7nAChR compound, GAT107, could be used to improve host defense functions in patients, such as those with COVID-19, who are exposed to prolonged periods of hyperoxia.

## 1. Introduction

Oxygen therapy, using concentrations of supplemental oxygen up to 100% (hyperoxia), is a routine treatment for intensive care units (ICU) patients, surgical patients, preterm neonates, patients with acute lung injury (ALI)/acute respiratory distress syndrome (ARDS), requiring home oxygen therapy, and in patients receiving supportive care for airway-associated infections, such as the novel COVID-19 coronavirus [1,2,3,4,5]. Although oxygen therapy is a life-saving intervention, prolonged exposure to hyperoxia can compromise lung host defense and cause acute lung injury, due to excessive inflammation (ALI) [6,7]. Consequently, patients with compromised host defenses that experience hyperoxia have a higher susceptibility to developing pulmonary bacterial infections that cause ventilator-associated pneumonia (VAP) and hospital-acquired pneumonia (HAP) [8,9]. Approximately one-third of all mechanically-ventilated (MV) ICU patients develop VAP, which has a 4.6% mortality rate [10,11,12].

Alveolar macrophages are the first line of defense against invading pathogens that enter the distal airways [13,14,15]. However, in mice and in ex vivo alveolar macrophage cultures, prolonged exposure to hyperoxia impairs the phagocytosis of bacteria known to produce VAP, such as *Staphylococcus aureus* and *Pseudomonas aeruginosa* (PA)*,* as well as certain bacteria from the *Enterobacteriaceae* family [8,16,17,18].

The prolonged exposure of macrophages to hyperoxia increases the levels of intracellular reactive oxygen species (ROS) [19], which can overwhelm the endogenous antioxidant defense system [19,20,21]. Consequently, high cytosolic levels of ROS can oxidize proteins and lipids, resulting in post-translational modification (PTM) of actin fibers [22,23]. Actin is a critical cytoskeletal component that is used by macrophages for chemotaxis and the formation of phagosomes around opsonized pathogens, such as PA [24]. Hyperoxia-induced actin oxidation contributes to the formation of stress fiber filaments, the dysregulation of actin polymerization, and the impairment of the migratory and phagocytotic functions of macrophages [22,23]. The impaired phagocytic functions of macrophages by hyperoxia-induced oxidative stress can be attenuated by activators of antioxidant defense pathways, such as sulforaphane or supplemental antioxidants, such as ascorbic acid, n-acetyl cysteine, and exogenous superoxide dismutase (SOD) [22,25]. Importantly, antioxidant molecules protect against the oxidation of actin monomer filaments, and restore the antibacterial and phagocytic functions of macrophages [22,23,26]. Thus, treatment with supplemental antioxidants or activators of antioxidant pathways could protect against the profound deleterious effects on macrophage-mediated bacterial killing in response to prolonged exposure to hyperoxia.

We and others have shown that the hyperoxia-compromised innate immune functions of alveolar macrophages are significantly attenuated by pharmacological activators of the α7 nicotinic acetylcholine receptors (α7nAChRs) [27,28]. The homomeric α7nAChR is a pentameric, ligand-gated ion channel located in both neuronal and non-neuronal cells [29,30,31]. Upon stimulation with endogenous acetylcholine, choline, or other cholinergic agonists, there is a rapid influx of calcium ions into neurons followed by activation of specific calcium-dependent pathways [29,32]. It has been reported that the cholinergic activation of non-neuronal cells, such as macrophages, produces an influx of calcium ions but does not significantly change whole-cell currents [33,34]. Moreover, several studies have demonstrated that the activation of peripheral α7nAChR produces anti-inflammatory effects [31,35,36]. It has been postulated that the anti-inflammatory efficacy produced by the activation of α7nAChR on peripheral immune cells, such as macrophages, may be due to: (1) inhibition of the phosphorylation of the transcription factor STAT3, which subsequently decreases inflammatory cytokine production [29]; (2) activation of the PI3K/Akt/Nrf2 antioxidant pathway and induction of heme oxygenase-1 (HO-1) or (3) the inhibition of NF-kB subunit phosphorylation and subsequent nuclear-translocation through STAT3-NF-kB convergence [29,32].

Previously, our lab has shown that GTS-21, a partial agonist of the α7nAChR, significantly increased bacterial clearance and decreased lung injury in hyperoxia-exposed mice with PA pneumonia [37]. Importantly, GTS-21 activates the cholinergic anti-inflammatory pathway, which has been shown to attenuate the phagocytic function of macrophages compromised by the prolonged exposure to hyperoxia [37]. However, other clinically relevant endpoints, such as mortality rates, were not significantly affected by GTS-21 administration (unpublished results). Nevertheless, it remains unclear if macrophage innate immune functions can be increased by attenuating oxidative stress via α7nAChR-dependent pathways.

Therefore, in this study, we conducted experiments to determine 1) the efficacy of the (+)—enantiomer of racemic 4-(4-bromophenyl)-3a,4,5,9b-tetrahydro-3H-cyclopenta[c]quinoline-8-sulfonamide, GAT107, to attenuate hyperoxia-induced impairment of host innate immune functions, and the mechanism of action of GAT107. GAT107 is a positive allosteric modulator (PAM) and direct allosteric activator (DAA) that (1) augments or potentiates the response to orthosteric site ligands and (2) activates the α7nAChR ion channel (in the absence of an orthosteric agonist) by binding to an allosteric site distinct from that of the PAM site [30,31,38,39,40]. In vitro, the α7nAChR can be rapidly desensitized by Ach. Interestingly, the combination of GAT107 with Ach produces a significant decrease in the Ach-induced desensitization [38,39]. GAT107 likely facilitates the conversion of desensitized states to conducting states, which surmounts receptor desensitization [38,39]. Therefore, these unique pharmacological properties of the novel α7nAChR ago-PAM, GAT107, may have advantageous efficacy in bacterial clearance and attenuating hyperoxia-compromised macrophage functions.

## 2. Materials and Methods

### 2.1. Cell Culture and Special Reagents

Murine macrophage-like RAW 264.7 cells (TIB-71; American Type Culture Collection (ATCC), Manassas, VA) were cultured in Dulbecco’s Modified Eagle Medium (DMEM) and supplemented with 10% FBS (Atlanta Biologicals, Lawrenceville, GA). Cells were maintained at 37 °C in normoxia (5% CO_2_/21% O_2_) for 24 h, allowed to grow to 70–80% confluency, and subcultured every 2 days. Bone marrow was harvested from 6 to 8-week-old male C57BL/6 mice (Jackson laboratories), isolated and cultured to allow for differentiation into bone marrow-derived macrophages (BMDM), as previously described (Weischenfeldt and Porse, 2008). Hyperoxic exposure was performed in sealed, humidified chambers (Billups-Rothenberg Inc., Del Mar, CA, USA) flushed with 95% O2/5% CO2 at 37 °C. An oxygen analyzer (MSA Medical Products, Pittsburgh, PA, USA) was used to monitor the O2 levels.

### 2.2. Animal Studies

Male C57BL/6 mice (6 to 10 weeks old; The Jackson Laboratory, Bar Harbor, ME, USA) were used in this study based on a protocol (protocol #1953) approved by the Institutional Animal Care and Use Committees at St. John’s University. The mice were housed in a specific pathogen-free environment, maintained at 22 °C (~50% relative humidity) with a 12 h light/dark cycle. All mice had *ad libitum* access to standard rodent food and water. Mice were randomized to receive either 3.3 mg/kg of ((+)—(4-(4-bromophenyl)-3a,4,5,9b-tetrahydro-3H-cyclopenta[c]quinoline-8-sulfonamide, GAT107 or saline, administered by intraperitoneal injection 24, 36, and 48 h after the onset of hyperoxic exposure. The dose of 3.3 mg/kg GAT107 was selected based on previous studies that it was safe and efficacious in ameliorating nociceptive-pain in mice [30]. After 48 h of exposure, the mice were inoculated with 0.1 × 10^8^ colony-forming units (CFUs) of PA by making a 1–2 cm incision on the neck to expose the trachea after anesthetization with sodium pentobarbital (75 mg/kg). PA was used as the selected pathogen as it is associated with 21% of all VAP cases [41]. Twenty-four hours after bacterial inoculation, mice were euthanized by exsanguination, and bronchoalveolar lavage (BAL) fluid was collected. Lung tissues were immediately collected into 1 mL cold PBS containing a protease and phosphatase inhibitors cocktail (Pierce Thermo Scientific) followed by homogenization by a Dounce tissue homogenizer, as described previously [42].

### 2.3. Exposure to Hyperoxia

Male C57BL/6 mice and cultured macrophages were exposed to hyperoxia, as previously described [42]. Briefly, animals were placed in microisolator cages (Allentown Caging Equipment, Allen-town, NJ, USA) that were kept in a Plexiglas chamber (BioSpherix, Lacona, NY, USA) and exposed to ≥95% O_2_ for up to 48 h. The exposure of murine macrophage RAW 264.7 cells was conducted in humidified Plexiglas chambers (Billups-Rothenberg, Del Mar, CA, USA), flushed with 95% O_2_/5% CO_2_ at 37 °C for 24 h. An oxygen analyzer (MSA; Ohio Medical Corporation, Gurnee, IL, USA) was used to monitor the O_2_ concentration in the chamber.

### 2.4. Bronchoalveolar Lavage

Murine BAL fluid was obtained, as previously described [42]. Briefly, mice were anesthetized by an intraperitoneal injection of sodium pentobarbital (75 mg/kg). Subsequently, a 1–2 cm incision was made on the neck, the trachea was dissected, and a 20-gauge × 1.25-inch intravenous catheter was inserted caudally into the lumen of the exposed trachea. The lungs were gently lavaged twice with 1 mL of a sterile, nonpyrogenic phosphate-buffered saline (PBS) solution (Mediatech, Herndon, VA, USA) containing a cocktail of protease and phosphatase inhibitors (Thermo Pierce Scientific). BAL samples were centrifuged at 200× *g* at 4 °C for 5 min, and the resultant supernatants were immediately used for quantitative bacteriology.

### 2.5. Quantitative Bacteriology

Viable bacterial counts were determined in serially-diluted LB (Luria-Bertani) broth lung homogenates and BAL fluid using a colony formation unit (CFU) assay by plating onto Pseudomonas Isolation Agar (Difco, Sparks, MD, USA) at 37 °C for 18 h.

### 2.6. Assay for Oxidative Stress and Antioxidant Potential

Oxidative stress was determined by measuring the oxidation-reduction potential (ORP) using the RedoxSYS Diagnostic System (Luoxis Diagnostics, Inc., Englewood, CO, USA). Lung homogenate was evaluated for its oxidative-reduction potential (ORP), reported in millivolts (mV), and the capacity of the ORP (cORP), also known as the antioxidant potential, was measured in microcoulombs (µC) at room temperature, using the protocol provided by the manufacturer.

### 2.7. Phagocytosis Assay and Actin Stress Filament Formation

The phagocytosis assay was performed, as previously described, with minor modifications [22,37]. Briefly, RAW 264.7 cells or BMDMs were seeded in 24-well plates and allowed to adhere for 6 h, followed by exposure to 95% O_2_ in the absence or presence of GAT107 (3.3 µM) for 24 h. The concentration of 3.3 µM of GAT107 was selected based on preliminary data obtained from phagocytosis assay studies. After 24 h, RAW 264.7 cells were incubated at 37 °C for 1 h with opsonized FITC-labeled latex beads (Polysciences, Warrington, PA, USA) at a ratio of 100:1 (beads:cell). Macrophages were incubated with 0.04% Trypan blue in PBS for 10 min to quench the beads that were not internalized by the macrophages. To visualize the uptake of FITC-labeled latex beads, macrophages were fixed with 4% paraformaldehyde for 10 min, washed with PBS, and stained with 4′,6-diamidino-2-phenylindole (DAPI, Molecular Probes, Eugene, OR, USA). To visualize the cell cytoskeleton, cells were stained with rhodamine phalloidin (Molecular Probes, Eugene, OR, USA). The phagocytosis or uptake of the beads was visually assessed using an Evos Fluorescent Microscope (Thermo Fischer, Waltham, MA, USA). The fluorescent beads in at least 200 individual macrophages per well, in duplicates, from three independent experiments were counted by an individual, blind to the experimental groups. To determine the hyperoxia-induced modification of actin polymerization, a modified experiment method was used [23]. Using the fluorescent micrographs obtained from the above phagocytosis assay, a Fiji ImageJ analysis (version 2.0) with a JACoP plugin, was used to determine Mander’s Correlation Coefficient using thresholds for the amount of phalloidin signal associated with the DAPI signal. This value was converted to a percentage to estimate the amount of polymerized F-actin and stress filament formation.

### 2.8. Measurement of SOD1 Activity

RAW 264.7 cells were seeded in 6-well plates, exposed to 95% O_2_, and incubated with 3.3 µM of GAT107 for 24 h. Subsequently, the cells were washed three times in PBS and lysed using a cell lysis buffer (Cell Signaling Technology, Danvers, MA, USA) supplemented with Halt protease and phosphatase inhibitors (Thermo Fischer, Waltham, MA, USA). The total protein content of cell lysate was determined using the Pierce Bicinchoninic acid (BCA) assay kit (Thermo Fisher, Waltham, MA, USA) as per the manufacturer’s instructions. Equal amounts of total protein from non-denatured samples were loaded onto 12% native-PAGE and separated at 100 V at 4 °C for 90 min. Native-PAGE gels were then washed three times with distilled water and then incubated (protected from light) with 2.43 mM of nitrotetrazolium blue (Acros Chemical), 2.85 µM of riboflavin (Sigma, St. Louis, MO, USA), and 28 mM of TEMED (GE Healthcare) for 20 min, protected from light and at room temperature, as previously described [43]. Next, the gels were placed on a lightbox and allowed to develop. The presence of achromatic bands represented the inhibition of nitrotetrazolium blue reduction by the SOD1 enzymatic activity, and the bands were detected using the Bio-Rad ChemiDoc imaging system (Bio-Rad, Hercules, CA, USA).

### 2.9. The Detection of Reactive Oxygen Species (ROS) Using 2’,7’-Dichlorofluorescin Diacetate (DCFH-DA)

RAW 264.7 cells were seeded in quadruplicate in 96-well black wall, clear bottom plates exposed to 95% O_2_, and incubated with 3.3 µM of GAT107 for 24 h. After 24 h, the level of ROS was determined using the 2’,7’-dichlorofluorescein diacetate (DCFH-DA) assay (Cell Biolabs, San Diego, CA, USA), according to the manufacturer’s instructions. In brief, the cells were incubated with 1 mM of DCFH-DA for 30 min at 37 °C and then were washed three times with warm PBS. The presence of total intracellular DCF (an indicator of ROS levels) was determined by lysing cells, as previously described [25], and the level of fluorescence was determined at 480/530 nm, using a Biotek Synergy LX multimode reader (Winooski, VT, USA). DCF relative fluorescent units were then reported as a percent relative to the 21% O_2_ exposure group (i.e., normoxic or room air control group).

### 2.10. Total Protein Oxidation Assay

The amount of total protein oxidation and carbonyl formation was determined using a commercially available Oxidized Protein Western Blot Kit (Abcam, Cambridge, UK), based on the manufacturer’s instructions. Equal amounts of whole-cell lysate from three to four independent experiments were exposed to protein carbonyl derivatization with 2,4-dinitrophenylhydrazone (DNP-hydrazone) in the presence of DNP-hydrazine, and unreacted samples were used as a negative control. The samples were separated by 12% SDS-PAGE and transferred to PVDF membranes. The membranes were washed and blocked in 5% milk/TBST solution. Next, the membranes were washed and incubated with secondary anti-DNP antibody, washed, and the immunoreactive DNP bands were developed enhanced chemiluminescence (ECL) reagents (Pierce Thermo Scientific, Waltham, MA, USA) and detected by the Bio-Rad ChemiDoc XRS+ imaging system (Biorad, Hercules, CA, USA).

### 2.11. Western Blot Analysis

For intracellular protein analysis, the cells were washed three times with PBS and lysed using a cell lysis buffer (Cell Signaling Technology, Danvers, MA, USA) supplemented with Halt and phosphatase inhibitor cocktail (Thermo Fischer, Waltham, MA, USA). The total protein content of cell lysate was determined by using the Bicinchoninic acid (BCA) assay kit (Thermo Fisher, Waltham, MA, USA), as per the manufacturer’s instructions. Samples were loaded onto 12% or 15% SDS-polyacrylamide gels (Bio-Rad, Hercules, CA, USA) and transferred to Immobilon-P membranes (Millipore, Bedford, MA, USA). Nonspecific binding sites on the membrane were blocked by incubating the membrane with 5% nonfat dry milk (Bio-Rad, Hercules, CA, USA) in Tris-buffered saline, containing 0.1% Tween 20 (TBST), for 1 h at room temperature. Next, the membranes were washed three times with TBST, and incubated overnight at 4 °C with anti-HO-1 (1:1000, #ab13248, Abcam, Cambridge, UK) and anti-pan-actin (1:1000, #8456, Cell Signaling) antibodies, diluted in 5% nonfat dry milk in TBST. After three washes with TBST, the membranes were incubated with goat anti-rabbit horseradish peroxidase-coupled secondary antibody (1:5000; GE Healthcare, Chicago, IL, USA) for 1 h at room temperature. Subsequently, membranes were again washed three times with TBST, and the immunoreactive proteins were visualized using the SuperSignal West Pico Plus Chemiluminescent Substrate (Thermo Fisher, Waltham, MA), as per the manufacturer’s instructions. Images were obtained using the Bio-Rad ChemiDoc XRS imaging system (Bio-Rad, Hercules, CA, USA). The immunoreactive bands were quantified using ImageJ software (version 2.0.0).

### 2.12. Nrf2 Activation Assay

Nrf2 activation was determined by measuring the amount of immunofluorescent Nrf2 nuclear co-localization. RAW 264.7 cells were seeded and prepared as described above for DCFH-DA assay. After 24 h, the cells were permeabilized in 0.1% Triton X-100/PBS (Millipore Sigma, St. Louis, MO, USA), blocked with 10% goat serum (Millipore Sigma) in PBS, and incubated with anti-Nrf2-antibody (donated by Dr. Edward Schmidt of Montana State University) diluted in 1% goat serum/PBS overnight. The cells were washed and immunoreacted with AlexaFluor488-conjugated secondary antibody (Pierce Thermo Scientific). Fluorescent micrographs were captured using an Evos fluorescent microscope. The images were analyzed for the amount of Nrf2 localized in the nucleus using a Mander’s Correlation Coefficient with thresholds as described above for the actin stress filament formation experiments. The amount of Nrf2 signal located within the same signal as the nucleus was used as a marker of Nrf2 activation. The Mander’s correlation coefficient was then reported as a percentage of the total Nrf2 signal within the nucleus.

### 2.13. Statistical Analysis

The statistical analyses were carried out using GraphPad Prism statistical software (version 7.0a). The results are presented as the mean ± SEM. All data sets were analyzed for statistical significance using analysis of variance (ANOVA) with Dunnett’s post-hoc analysis. A 95% confidence interval was used for all data sets and the *a priori* significance level was *p* < 0.05 (* *p* < 0.05; ** *p* < 0.01; *** *p* < 0.001; **** *p* < 0.0001).

## 3. Results

### 3.1. The Systemic Administration of GAT107 Increases Bacterial Clearance and Attenuates the Hyperoxia-Induced Redox Imbalance in Mice Challenged with Pseudomonas Aeruginosa (PA)

**Figure 1:**
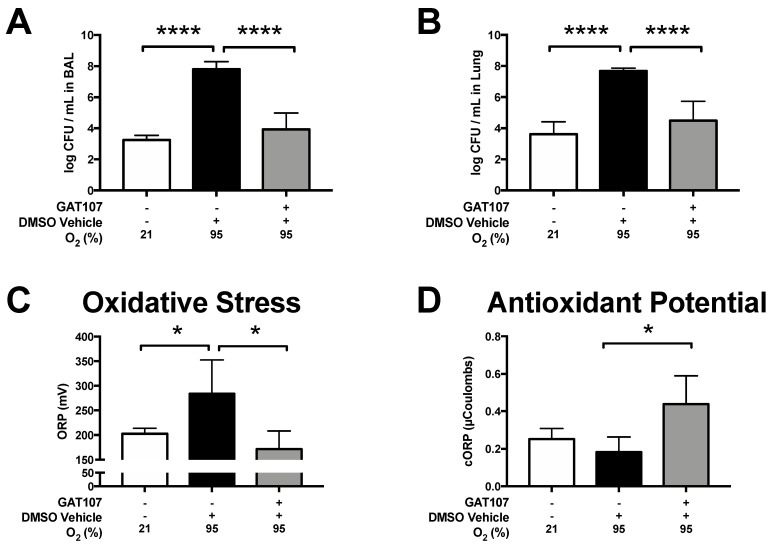
The intraperitoneal administration of GAT107 increases bacterial clearance and attenuates the hyperoxia-induced redox imbalance in mice challenged with intra-tracheal *Pseudomonas aeruginosa.* Mice exposed to >95% O_2_ (hyperoxia) for 48 h, inoculated with PA (0.1 × 10^8^ CFUs/mouse) and then returned to 21% O_2_ after inoculation. Mice were randomized to receive 3.3 mg/kg of GAT107 or vehicle intraperitoneally, every 12 h, starting after 24 h of exposure to hyperoxia. Bronchoalveolar lavage (BAL) and lung tissue were harvested 24 h after inoculation. Viable bacteria in the airways and lungs were quantified by plating serials dilutions of (**A**) BAL and (**B**) lung homogenate and were expressed as the log of colony-forming units (CFUs) per mL. Data represent the mean ± SEM based on *n* = 6–7 mice per group. Statistical differences were determined between all groups and indicated as **** *p* < 0.0001 compared to the hyperoxia-exposed group treated with vehicle (i.e., the control group). Lung homogenate was analyzed for (**C**) oxidative-reduction potential (ORP), expressed as millivolts (mV) and (**D**) the capacity of the ORP or total antioxidant potential (cORP), expressed as µCoulombs (µC), by the RedoxSys System. Data represent the mean ± SEM of two-independent experiments based on *n* = 4 mice per group. Statistical differences were determined between all groups and indicated as * *p* < 0.05 compared to the hyperoxia-exposed group treated with vehicle.

Prolonged exposure to hyperoxia can compromise the clearance of pathogenic bacteria in the lungs. [37,42,44]. To determine if GAT107 can restore the hyperoxia-compromised clearance of bacteria in the lungs, mice were continuously exposed for 48 h to 95% O_2_ (i.e., hyperoxia), administered intraperitoneal (i.p.) injections of 3.3 mg/kg GAT107 at 24, 36, and 48 h of hyperoxia exposure, and inoculated with intra-tracheal (i.t.) injection of PA at the end of 48 h of hyperoxia exposure. Twenty-four hours post-i.t. inoculation of PA, bronchoalveolar lavage fluids, and lung tissue homogenate were collected and analyzed for their bacterial counts using a colony formation unit assay. As shown in Figure 1A,B, mice that received prolonged exposure to hyperoxia and were challenged with PA-induced lung infection (vehicle control group), had significantly higher levels of bacterial colonies in their airways (7.81 ± 0.24 log CFUs/mL, Figure 1A *p* < 0.0001) when compared to mice that remained at 21% O_2_ (3.24 ± 0.15 log CFUs/mL). Additionally, mice exposed to hyperoxic conditions also had a significantly higher number of bacterial counts in the lung tissue homogenate (7.68 ± 0.07 log CFUs/mL, Figure 1B, *p* < 0.0001) compared to mice that remained at 21% O_2_ (3.62 ± 0.35 log CFUs/mL in lung tissue homogenate). The mice that received 3.3 mg/kg i.p. of GAT107 had significantly decreased levels of bacteria in their airways (3.93 ± 0.52 CFUs/mL in airways, Figure 1A) compared to vehicle control group. The administration of 3.3 mg/kg i.p GAT107 also significantly attenuated the level of bacteria in the lung tissue homogenate (4.49 ± 0.54 log CFUs/mL in lung tissue homogenate, Figure 1B), compared to the vehicle control group (**** *p* < 0.0001). No statistical differences in the levels of bacterial colonies in BAL fluid or lung tissue homogenate were observed between GAT107-treated animals and those exposed to room air (Figure 1A,B).

Previous studies have shown that hyperoxia-induced oxidative stress mediates the decreased bacterial clearance functions in mice, which can be attenuated by certain antioxidants, such as ascorbic acid [44]. To determine if GAT107 decreases excessive lung oxidative stress in mice exposed to 95% O_2_ (hyperoxia) and challenged with PA lung infection, the oxidative redox potential (ORP), and the lung antioxidant potentials (cORP) were determined. As shown in Figure 1C and D, mice exposed to 95% O_2_ had significantly higher oxidative-reduction potentials (298 ± 44.3 mV, *p* < 0.05) and lower antioxidant potentials (0.16 ± 0.05 µC, *p* < 0.05), compared to mice that remained at 21% O_2_ (204.26 ± 7.5 mV and 0.27 ± 0.03 µC, respectively). The administration of 3.3 mg/kg i.p. of GAT107 significantly attenuated the hyperoxia-induced increased lung oxidative-reduction potential (162.36 ± 22.1 mV, *p* < 0.05) and increased the total antioxidant potential (0.47 ± 0.09 µC, *p* < 0.05), compared to the hyperoxic vehicle control group (Figure 1C,D). Lung homogenate ORP and cORP levels were not statistically different from animals that remained in room air conditions. These data indicate that GAT107 significantly decreases the bacterial lung burden by decreasing lung oxidative stress and increasing the total antioxidant potential in a mouse model of VAP.

### 3.2. GAT107 Restores Hyperoxia-Compromised Macrophage Phagocytic Function in a Macrophage Cell Line and Primary Macrophages

**Figure 2:**
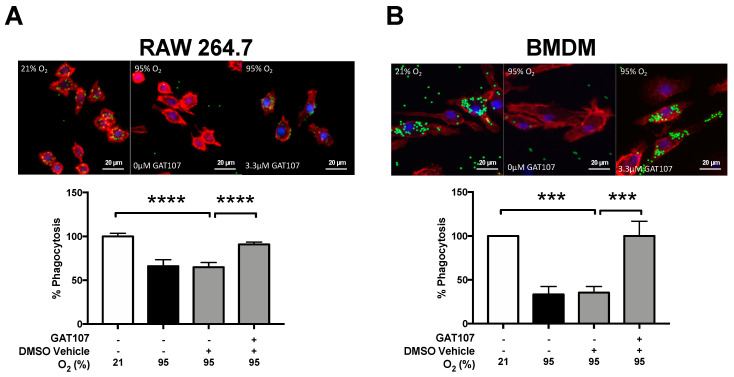
GAT107 restores hyperoxia-compromised macrophage phagocytic function in RAW 264.7 cells and primary macrophages. RAW 264.7 cells were either exposed to 21% O_2_ (white bar) or 95% O_2_ (hyperoxia) (black bar) in the presence or absence of 3.3 µM GAT107 (grey bars). Cells were incubated with FITC-labeled minibeads for 1 h and stained to visualize the cytoskeleton and nucleus. Immunofluorescent micrographs show the phagocytosed beads (green), cytoskeleton (red), and nucleus (blue) of (**A**) RAW 264.7 cells and (B) BMDMs. The bar graphs represent the percentage of beads phagocytosed (A) RAW 264.7 and (**B**) BMDMs cells, quantified from at least 200 cells per group. Each value represents the mean ± SEM of three independent experiments for each group. Statistical differences were determined between all groups and indicated as **** *p* < 0.0001, *** *p* < 0.001 compared to the hyperoxia-exposed vehicle control group.

The decreased in the clearance of bacterial lung infections due to hyperoxia, is, in part, mediated by the impairment of macrophage immune function [22,26,42]. To determine whether GAT107 can restore hyperoxia-compromised macrophage function, RAW 264.7 and primary bone marrow-derived macrophages (BMDMs) were exposed to 95% O_2_ (hyperoxia) for 24 h and incubated with either 3.3 µM of GAT107 or vehicle control (DMSO). The phagocytotic function of RAW 264.7 cells exposed to hyperoxia was significantly decreased (66.1 ± 2.5%, *p* < 0.0001) compared to cells that remained at 21% O_2_ (100 ± 0%) (Figure 2A). Hyperoxia-compromised phagocytosis was significantly attenuated by 3.3 µM of GAT107 (86.3 ± 4.7%, *p* < 0.0001), compared to the vehicle control group (65.08 ± 2.53%) (Figure 2A). Furthermore, these effects were also replicated in primary BMDMs, where after exposure to 24 h of hyperoxia, their phagocytic activity was significantly decreased (33.2 ± 5.3%, *p* < 0.0001), compared to macrophages that remained at 21% O_2_ (100 ± 0%). GAT107 significantly increased the phagocytotic activity of BMDMs exposed to hyperoxia (105 ± 13.9%, *p* < 0.001), compared to the vehicle control group (35.39 ± 3.9%) (Figure 2B). The incubation of hyperoxia-compromised macrophages with 3.3 µM of GAT107 significantly increased their phagocytotic activity to a level that was not statistically different compared to macrophages exposed to room air. These in vitro results suggest that in vitro, GAT107 can rescue the hyperoxic-induced decrease in macrophage phagocytic activity.

### 3.3. GAT107 Decreases Hyperoxia-Induced Actin Oxidation and Alterations in Polymerization

**Figure 3:**
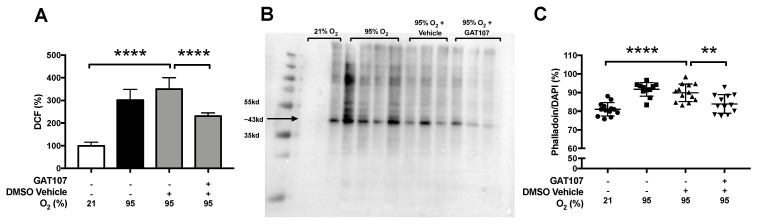
GAT107 attenuates hyperoxia-induced oxidative stress and decreases hyperoxia-induced actin oxidation and hyper-polymerization of actin in macrophages. RAW 264.7 were either exposed to 21% O_2_ or 95% O_2_ (hyperoxia) in the presence or absence of 3.3 µM GAT107. After 24 h of hyperoxic exposure (**A**), the fluorescent DCF signal of the macrophages was determined using the DCFH-DA assay and was quantitated spectrophotometrically and reported as a percent relative to the room air control to evaluate oxidative stress. (**B**) The total protein carbonylation, due to oxidation, was determined using the western blot assay. The 43kD immunoreactive band was used to evaluate the level of actin oxidation (as indicated by the arrow) based on 3–4 independent experiments per group. (**C**) The amount of actin polymerization was determined using the fluorescent micrographs obtained as described in Section 2.7, and the ratio of the occupied area of actin relative to the area of the nuclei, was reported as a percent of non-overlapping signals—obtained from ImageJ’s co-localization analysis plugin and computation of Manders’ Correlation Coefficient. Each value represents the mean ± SEM of three independent experiments for each group. Statistical differences were determined between all groups and indicated as **** *p* < 0.0001, ** *p* < 0.01 compared to the hyperoxia-exposed vehicle control group.

To determine if hyperoxia-induced oxidative stress in macrophages is affected by GAT107, RAW 264.7 cells were exposed >95% O_2_ (hyperoxia) and incubated with 3.3 µM of GAT107, the vehicle control (DMSO), or untreated macrophages. Intracellular ROS levels, a determinant of oxidative stress, were measured and compared to room air control cells. The prolonged exposure to hyperoxia significantly increased the total intracellular ROS levels (305.79 ± 20.21%, **** *p* < 0.0001), compared to macrophages that remained at 21% O_2_ (100 ± 0%) (Figure 3A). Incubation with GAT107 significantly decreased the total intracellular ROS levels (231.05 ± 5.75%, **** *p* < 0.0001), compared to vehicle control (353.28 ± 27.81%) (Figure 3A).

Macrophages exposed to hyperoxia can produce oxidative post-translational modifications of the actin filaments that are critical for the innate immune functions of macrophages [22,23]. To determine if GAT107 attenuates hyperoxia-induced actin oxidation and alters actin polymerization, RAW 264.7 macrophages were exposed to 24 h of 95% O_2_ (hyperoxia) and incubated with 3.3 µM of GAT107. The fluorescent microscopic analysis and quantification of F-actin stress fiber formation, similar to previous studies [22,23], indicated that untreated macrophages exposed to hyperoxia had significantly higher amounts of stress actin filament formation, as indicated by an increase in the phalloidin/DAPI ratio (91.25 ± 1.09%, **** *p* < 0.0001), compared to cells that remained at room air (81.05 ± 1.07%)(Figure 3B). Furthermore, 3.3 µM of GAT107 significantly decreased stress actin formation produced by hyperoxia (83.45 ± 1.53%, *p* < 0.01), compared to the vehicle control group (89.53 ± 1.34%) (Figure 3B). Stress fiber formation in GAT107-treated, hyperoxia compromised macrophages was not statistically different from cells that remained in room air control conditions (Figure 3B). Total protein oxidation was also assessed in macrophages exposed to hyperoxia for 24 h. Hyperoxia increased the total protein oxidation in macrophages compared to the macrophages that were exposed to room air (Figure 3C). This augmentation was decreased by incubation with 3.3 µM of GAT107 (Figure 3C).

The protein samples that migrated to a band, which was approximately 43 kD, were used as an indicator of actin oxidation. The 43 kD band was chosen due to the electrophoretic migration of monomeric actin by SDS-PAGE, as previously demonstrated by O’Reilly et al. 2003. The incubation of cells with 3.3 μM of GAT107 decreased the observable response to hyperoxia at the level of the 43 kD marker, suggesting that GAT107 decreased the amount of hyperoxia-induced actin oxidation. These results indicate that GAT107 decreases oxidative stress and hyperoxia-induced alterations in actin polymerization by decreasing the oxidation of total protein and actin.

### 3.4. GAT107 Restores Hyperoxia-Compromised SOD1 Function

**Figure 4:**
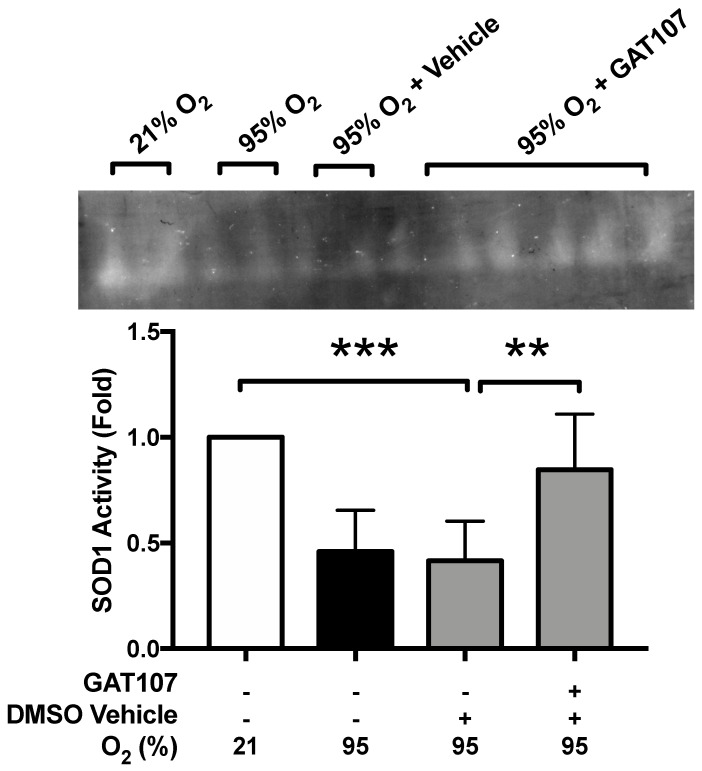
GAT107 restores superoxide dismutase 1 activity in hyperoxia-compromised macrophages. RAW 264.7 cells were either exposed to 21% O_2_ (room air) (white bar) or 95% O_2_ (hyperoxia) (black bar) in the presence or absence of 3.3 µM GAT107 (grey bars). After 24 h of exposure to hyperoxia, SOD1 activity was determined using the gel-based nitro tetrazolium blue gel assay, as described in Section 2, and was reported as a fold-change compared to the room air control group. Each value represents the mean ± SEM of three independent experiments for each group. Statistical differences were determined between all groups and indicated as *** *p* < 0.001 or ** *p* < 0.01 compared hyperoxia-exposed group treated with vehicle.

The prolonged exposure to hyperoxia induces oxidative stress, which compromises the innate immune functions of macrophages, which is attenuated by antioxidants, such as ascorbic acid and superoxide dismutase (SOD) [22,25,26]. After 24 h exposure to 95% O_2,_ (hyperoxia), the levels of SOD1 activity were assessed in RAW 264.7 cells. Prolonged hyperoxia significantly decreased SOD1 activity in the cultured macrophages (45.59 ± 8.71%, *** *p* < 0.001), compared to RAW 264.7 macrophages that remained at 21% O_2_ (100 ± 0%) (Figure 4). The incubation of macrophages exposed to hyperoxia with 3.3 μM of GAT107 significantly increased SOD1 activity (84.63 ± 10.75%, ** *p* < 0.01), compared to the vehicle control (41.56 ± 7.31) (Figure 4). These results suggest that hyperoxia-induced oxidative stress is decreased by GAT107, in part, by increasing the antioxidant activity of SOD1 in macrophages exposed to hyperoxia.

### 3.5. GAT107 Activates the Nrf2/HO-1 Antioxidant Pathway

**Figure 5:**
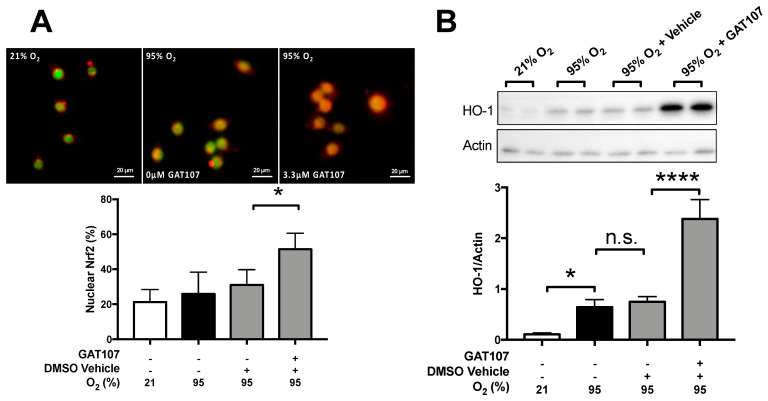
GAT107 Activates the Nrf2/HO-1 antioxidant pathway. RAW 264.7 cells were either exposed to 21% O_2_ (white bar) or 95% O_2_ (hyperoxia) (black bar) with or without GAT107 (grey bars). After 24 h of exposure to hyperoxia, macrophages were fixed, permeabilized, blocked, incubated with a polyclonal anti-Nrf2-antibody overnight, and then conjugated to AlexaFluor 488. Finally, the slides were counterstained with DAPI to visualize the nucleus. (**A**) Immunofluorescent micrographs were subjected to ImageJ co-localization analysis and re-pseudo colored to determine the amount of Nrf2 signal (red) localized to the nucleus (green) using Manders’ Correlation Coefficient. (**A**) The bar graphs represent the percent amount of total Nrf2 signal localized to the nuclear region. Under the same experimental conditions, the levels of heme oxygenase-1 (HO-1) were determined in macrophages, and the levels of actin in whole-cell lysate were determined using the western blot assay. (**B**) Representative immunoreactive bands for HO-1 and actin and (**B**) the quantification of immunoreactive bands normalized to actin. Each value represents the mean ± SEM of two to three independent experiments for each group. Statistical differences were determined between all groups and indicated as **** *p* < 0.0001, * *p* < 0.05, n.s. = non-significant compared to the hyperoxia-exposed group treated with vehicle.

Previous studies indicate that the activation of Nrf2 can protect mice from hyperoxia-induced acute lung injury and macrophage dysfunction by upregulating enzymes involved in the antioxidant pathways, such as heme oxygenase-1 (HO-1) [45,46,47]. To determine if GAT107 can induce the activation of Nrf2 and nuclear localization of Nrf2, macrophages were exposed to 24 h of 95% O_2_ (hyperoxia) and incubated with 3.3 µM of GAT107 or DMSO vehicle. Under hyperoxic conditions, there was no significant increase in Nrf2 localization to the nucleus (25.83 ± 7.19%), compared to macrophages exposed to room air (25.83 ± 7.19% of cells versus 21.2 ± 4.8% of cells with nuclear Nrf2, respectively) (Figure 5A). However, GAT107 significantly increased Nrf2 nuclear localization (51.45 ± 4.08% versus 31.03 ± 5.01% of cells, respectively, * *p* < 0.05), compared to vehicle control (31.03 ± 5.01%) (Figure 5A). Next, the levels of HO-1 protein in macrophages were determined using western blot analysis. Under hyperoxic conditions, there was also a significant increase (*p* < 0.05) in HO-1 (0.643 ± 0.075 AU HO-1/actin) levels, compared to macrophages exposed to 21% O_2_ room air (0.107 ± 0.01 AU HO-1/actin) (Figure 5B). However, the incubation of macrophages with 3.3 µM of GAT107 (2.38 ± 1.91 AU HO-1/actin, **** *p* < 0.0001) induced a significant increase in HO-1 levels, compared to the vehicle control (0.745 ± 0.05 AU HO-1/actin) (Figure 5B). These in vitro results suggest thatGAT107 induces antioxidant pathways in macrophages through Nrf2 activation and upregulation of HO-1 protein levels.

## 4. Discussion

This study demonstrates that GAT107 activates α7nAChR-dependent pathways within the neuromodulated cholinergic anti-inflammatory system and GAT107 attenuated oxidative stress by increasing the antioxidant response. The administration of 3.3 mg/kg i.p. of GAT107 to mice significantly increased bacterial clearance by decreasing the level of clinically relevant oxidative stress markers in the lung. Moreover, the protective effects of GAT107, due to the increase in the clearance of *P. aeruginosa*, coincided with the restoration of hyperoxia-compromised macrophage functions. The impairment of macrophage function was attenuated by the GAT107-mediated decrease in the total protein oxidation, including the oxidation of actin, a critical cytoskeletal component involved in macrophage phagocytosis. Furthermore, GAT107 increased the activity of the antioxidant enzyme, SOD1, and activated the protein transcription factor, Nrf2, and upregulated the levels of the downstream mediator, HO-1. Taken together, these results suggest that GAT107 increases bacterial clearance by increasing the phagocytic function of macrophages exposed to hyperoxia by increasing macrophage redox. 

### 4.1. GAT107-Mediated Attenuation of Oxidative Stress Contributes to the Attenuation of Hyperoxia-Compromised Bacterial Clearance Functions of Mice with PA Lung Infection

It is known that high levels of PA in the lungs can damage pulmonary tissue, resulting in increased mortality rates in ventilated patients [48,49,50]. As shown in Figure 1, mice exposed to hyperoxia and challenged with intra-tracheal PA, have an impaired capacity to clear PA in both the airways and in lung tissue. The administration of GAT107 significantly attenuated the host defense response compromised by hyperoxia. Compared to our previous findings, where the administration of 4 mg/kg i.p. of GTS-21 three times a day under the same experimental paradigm utilized in this study [37], also attenuated hyperoxia-compromised bacterial clearance in mice. In contrast, the twice-daily administration of 3.3 mg/kg i.p. of GAT107 to mice exposed to hyperoxia produced a ten-fold increase in the clearance of bacteria. Since α7nAChRs are susceptible to receptor desensitization [31], we hypothesized that the unique ago-PAM properties of GAT107 might allow for lower and less frequent dosing, thereby producing a similar magnitude of bacterial clearance, although a more detailed pharmacological evaluation will be required to verify this hypothesis. Nevertheless, these results, provided that they can be translated to humans, suggest that GAT107 could improve clinically relevant outcomes in patients receiving oxygen therapy for extended periods by increasing the innate immune function of macrophages, thereby decreasing the incidence of hospital-acquired infections [51,52,53,54]. It is important to note that direct vagus nerve stimulation, which activates α7nAChRs, increases the survival rates of subjects with sepsis [55,56,57,58]. Interestingly, other studies have indicated that the inhibition of α7nAChR with antagonists (methyllycaconitine and α-bungarotoxin), only partially reduces GTS-21’s efficacy to inhibit LPS-induced secretion of IL-6 and TNFα [59]. GTS-21 also partially ameliorates LPS-induced secretion of both IL6 and TNFα from cultured macrophages where the α7nAChR gene was knocked out [59]. In our study, we did not determine whether the efficacy of GAT107 was due to its selective activation of the α7nAChR. Thus, it is possible that GAT107 may interact with non-α7nAChR targets, and future studies will be required to identify these targets. Our results indicated that the activation of α7nAChR-dependent pathways with GAT107 in mice produced its efficacy by modulated clinically relevant parameters of oxidative stress. As shown in Figure 1C and D, the i.p. administration of 3.3 mg/kg GAT107 significantly decreased the hyperoxia-induced increases in ORP elevation in mice by increasing the total antioxidant potential in the lung tissue homogenate. This is important as an increase in oxidative stress can produce severe illness and death. For example, in a clinical trial involving 645 people with traumatic brain injury, a 20 mV increase in plasma ORP levels was positively correlated with a 4-fold increase in mortality [60]. Conversely, a 1 unit increase in 1/cORP (antioxidant potential) was positively correlated with a 5-fold increase in mortality [60]. Recently, we have reported that in mice exposed to 72 h of hyperoxia, there was a significant increase in lung lavage fluid ORP levels [25]. Furthermore, 24 h of hyperoxia exposure induced a significant increase in cultured macrophage lysate ORP levels [44]. Furthermore, in both of the aforementioned studies, ascorbic acid (50 mg/kg i.p. or 1000 µM in cell culture) significantly decreased the hyperoxia-induced increase in lysate ORP levels in mouse lung lavage fluid and cultured macrophages levels, and the mortality rate was significantly diminished following the intra-tracheal administration of *P. aeruginosa* [25,44].

Recently, it has been reported that the major cause of death in people with COVID-19 is due to pneumonia, which is in part mediated by a hyper-inflammatory response [61]. It has been posited that the use of non-invasive vagus nerve stimulation, which activates the anti-inflammatory α7nAChR-mediated response, may be a potential treatment strategy to decrease the hyper-inflammatory syndrome observed in COVID-19 [62,63]. Thus, the identification of novel therapeutic strategies that provide protection against hyper-inflammation and oxidative stress (caused by COVID-19 or clinical supplemental oxygen therapy), such as activators of the cholinergic anti-inflammatory pathway (e.g., GAT107, GTS-21 and vagus nerve stimulation), could restore critical host innate immune function and decrease the severity and incidence of pneumonia.

### 4.2. GAT107 Significantly Attenuates Impaired Macrophage Innate Immune Functions Produced by Hyperoxia by Decreasing the Oxidation of Actin

Our results demonstrated that 3.3 μM of GAT107 significantly attenuated the hyperoxia-induced impairment of macrophage phagocytic function in cultured macrophages and primary BMDMs (Figure 2). Previously, we and others have reported that prolonged exposure to hyperoxia decreases macrophage phagocytic function [22,37,42,64]. The partial-α7nAChR agonist, GTS-21 (5–50 µM), decreases impaired phagocytic function produced by hyperoxia, which was due, in part, to a decrease in HMGB1 release from macrophages [37]. The GTS-21-mediated decrease in airway HMGB1 also increased bacterial clearance in the airways of mice challenged with intra-tracheal PA infection [37]. Similar to our bacterial clearance results (Figure 1), GTS-21, at 25–50 µM [37], and GAT107, at 3.3 µM, had similar efficacy in significantly attenuating hyperoxia-induced phagocytic dysfunction of cultured macrophages. Thus, the ago-PAM properties of GAT107, may produce greater efficacy than the partial-agonism of α7nAChR by GTS-21. In addition, the direct stimulation of the vagus nerve has been previously shown to increase the basal and sepsis-challenged phagocytic activity of resident liver macrophages [65], suggesting that the activation of α7nAChR in macrophages may play a critical role in modulating intracellular pathways that mediate phagocytic activity.

In this study, the exposure of macrophages to hyperoxia produced oxidation of proteins that caused the disorganization of actin fibers. These results are consistent with previous studies reporting that the prolonged exposure to hyperoxia and intra-tracheal PA oxidizes actin filaments, resulting in dysfunctional actin polymerization [22,23]. This is important as macrophage phagocytic function is dependent upon the rapid polymerization of actin filaments for migration and phagocytosis of bacteria [66]. Protein oxidation of actin filaments may be due to macrophage activation, which increases superoxide production during the oxidative or respiratory burst response [67]. The respiratory burst response involves the assembly of NADPH oxidase enzymes that generate large amounts of superoxide, which kill the ingested pathogens [67]. However, macrophages exposed to hyperoxia for prolonged periods have significantly increased intracellular levels of superoxide [19,20,21]. Consequently, hyperoxia exposure, in addition to activating respiratory bursts, may produce excessive oxidative stress, where high levels of ROS can induce the post-translational oxidation modifications of proteins, such as actin [68,69,70].

In this study, 3.3 µM of GAT107 significantly decreased the hyperoxia-induced actin fiber disorganization in macrophages by decreasing the magnitude of protein oxidation. This result is consistent with a study reporting that the activation of neutrophil α7nAChRs with the direct agonist, nicotine, decreases the polymerization of actin [71]. In addition, antioxidants, such as procysteine and exogenous superoxide dismutase (SOD), protect against hyperoxia-induced disorganization of the actin cytoskeleton, and restore phagocytic dysfunction [22]. Furthermore, hyperoxia-compromised macrophages have a lower bactericidal capacity, which may be partly due to actin disorganization and subsequent dysfunction of the assemble of bactericidal enzymes or phagolysosomes [72]. Overall, our results suggest that the efficacy of GAT107 to attenuate hyperoxia-compromised macrophage function may be due, in part, to it decreasing the oxidation of actin filaments.

### 4.3. GAT107 Decreases Oxidative Stress and Restores Antioxidant Functions

As shown in Figure 3, the prolonged exposure to hyperoxia increased total ROS levels in macrophages and significantly decreased the antioxidant activity of SOD1. Our results indicated that GAT107 significantly decreased the hyperoxia-induced increase in macrophage ROS levels, as well as increasing SOD1 activity to the level of that in cells incubated with vehicle (Figure 4). We hypothesize that the activation of the α7nAChR by GAT107 could restore hyperoxia-compromised SOD1 activity by transcriptional upregulation and expression of SOD1 or by altering inhibitory post-translational regulatory modifications of SOD1 [73]. Decreased SOD1 activity during hyperoxic conditions could further increase the levels of cytoplasmic superoxide. Thus, high cytoplasmic ROS levels, in addition to a compromised antioxidant defense system, may produce further oxidation of macromolecules, such as F-actin. Indeed, biochemical analysis and computational modeling suggest that NADPH oxidase (NOX)-mediated superoxide production causes the oxidation of actin filaments at Cys10, 217, 257, 285, and 374 [74].

### 4.4. GAT107 Activates Nrf2 and Upregulates HO-1

The GAT107-mediated restoration of the hyperoxia-induced redox imbalance may be due to its activation of the master antioxidant pathway component, Nrf2 (Figure 5A). Recently, we and others have shown that the activation of the Nrf2 pathway in macrophages in vitro and ex vivo in mice significantly increases innate immune function and decreases lung injury [25,46,75]. The activation of α7nAChR by Ach or GTS-21 results in downstream Nrf2 activation and the transcriptional upregulation of the genes code for antioxidant molecules, such as glutathione [21,32,76,77,78]. Heme oxygenase-1 (HO-1) is an Nrf2-regulated and the rate-limiting enzyme in heme metabolism, which has been shown to decrease oxidative-induced lung injury in mice [46,75,79,80]. Under hyperoxic conditions, HO-1 null cardiomyocytes had mitochondrial damage and decreased density, which may be due to heme toxicity [81]. Furthermore, hyperoxia exposure increases the number of hemoproteins and free reactive iron in the lung tissues of mice [82]. It is likely that the free iron and the heme groups in hyperoxia are derived from cytochromes, cyclooxygenases, and other heme-containing proteins present in the mitochondria [83].

In HO-1-deficient mice with sepsis, there was a decrease in bacterial clearance by macrophages, and this was attenuated by pretreating animals with carbon monoxide-releasing molecules [84]. Conversely, in mice exposed to hyperoxia with disruption or inhibition of HO-1 expression, the levels of total reactive iron in the lung were decreased and the magnitude of inflammatory lung injury was decreased [85]. Although the exact role that HO-1 plays in the lungs of mice and macrophages exposed to hyperoxia is not fully understood, the early induction of HO-1 by GAT107 could produce an antioxidant effect that attenuates hyperoxia-induced lung injury and increases bacterial clearance by macrophages.

## 5. Conclusions

**Figure 6:**
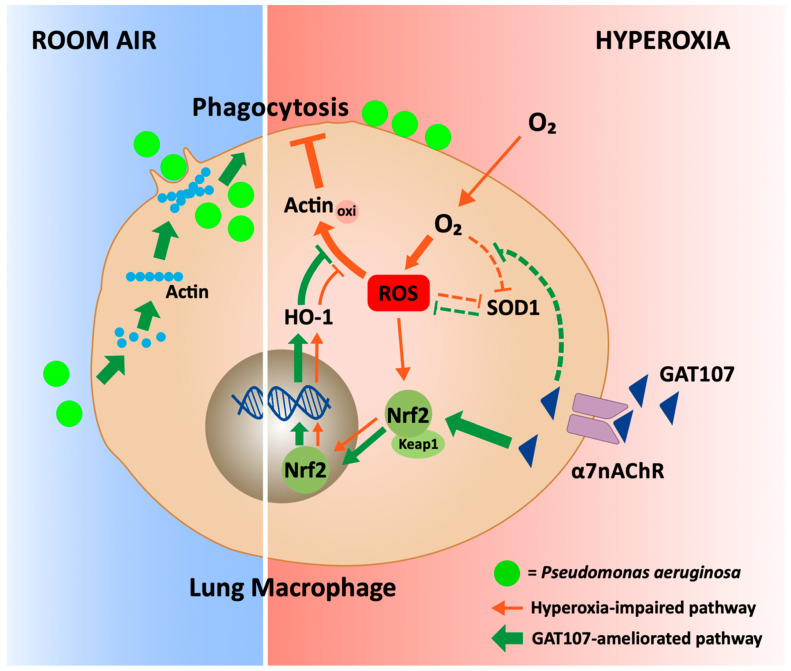
The proposed mechanism by which GAT107 restores bacterial clearance in the lungs by reducing oxidative stress, due to hyperoxia. Under room air conditions, *Pseudomonas aeruginosa* (PA) infection facilitates the re-organization of the F-actin cytoskeleton, thereby facilitating bacterial clearance by macrophage phagocytosis. The exposure of macrophages to hyperoxia increases ROS levels, which overwhelms the antioxidant response, resulting in oxidative stress (Morrow et al., 2007; Patel et al., 2016). Oxidative stress oxidizes actin, altering the formation of F-actin and impairing the phagocytic activity involved in bacterial clearance in the lungs. The α7nAChR ago-PAM, GAT107, significantly restores hyperoxia-compromised phagocytic activity by inhibiting the oxidation of actin by attenuating the hyperoxia-induced decrease in SOD1 activity and the upregulating HO-1 via the Keap1/Nrf2 antioxidative pathway. These GAT107-mediated effects reverse the hyperoxia-induced impairment of macrophage phagocytosis.

As shown in Figure 6, the α7nAChR type 2 ago-PAM, GAT107, attenuates hyperoxia-induced dysfunction of bacterial clearance in mice inoculated with *P. aeruginosa* via intra-tracheal administration. GAT107 restored the clearance of *P. aeruginosa* in mice, in part, by inducing an antioxidant response in the lungs, thereby decreasing oxidative stress. Furthermore, GAT107 was efficacious in attenuating macrophage phagocytic dysfunction induced by hyperoxia. GAT107 also attenuated the increase in ROS levels caused in macrophages caused by hyperoxia. GAT107’s attenuation of macrophage oxidative stress played a role in decreasing hyperoxia-induced oxidization of total protein and F-actin filaments in macrophages. Indeed, in macrophages, GAT107 decreased the significant increase in intracellular ROS levels and loss of SOD1 antioxidant function produced by hyperoxia. GAT107 also activated Nrf2 and upregulated HO-1 expression, which would decrease hyperoxia-induced oxidative stress. GAT107 attenuates hyperoxia-compromised bacterial clearance in mice by attenuating the redox imbalance in macrophages. Thus, the rapid development of a7nAChR agonists, such as GTS-21 and GAT107, which has already been evaluated for the treatment of certain neurodegenerative diseases, could be beneficial for patients with pulmonary infections, such as VAP and COVID-19 [86].

In conclusion, our results, provided they can be extrapolated to humans, suggest that GAT107 may be a potential therapeutic candidate that may prevent or treat patients exposed to high levels of oxygen for a prolonged period of time.

## Data Availability

Data available upon reasonable request.
